# Enhanced Recombinant Protein Expression in Insect Cells by Natural and Recombinant Components of Lepidoptera Hemolymph

**DOI:** 10.3390/v16060944

**Published:** 2024-06-12

**Authors:** Javier López-Vidal, Susana Martínez-Pulgarín, Diego Martínez-Alonso, Miguel Cid, José M. Escribano

**Affiliations:** 1 Alternative Gene Expression S.L (ALGENEX), Ronda de Poniente 14, Tres Cantos, 28760 Madrid, Spain; javierlopezvidal1@gmail.com (J.L.-V.); susana.martinezp@algenex.com (S.M.-P.); miguelcidf@gmail.com (M.C.); 2 Department of Cancer Biology, Dana-Farber Cancer Institute, Boston, MA 02215, USA; diego_martinezalonso@dfci.harvard.edu

**Keywords:** baculovirus, *Trichoplusia ni*, recombinant protein, hemolymph, *Bombyx mori*, protein SP2Bm

## Abstract

Prior research has established the anti-apoptotic effects in insect cell cultures of *Bombyx mori* (*B. mori*) hemolymph, as well as the heightened production yields of recombinant proteins facilitated by baculovirus vectors in insect cells cultivated in media supplemented with this hemolymph. In this study, we investigated the hemolymph of another Lepidoptera species, *Trichoplusia ni* (*T. ni*), and observed similar beneficial effects in insect cells cultivated in media supplemented with this natural substance. We observed enhancements in both production yield (approximately 1.5 times higher) and late-stage cell viabilities post-infection (30–40% higher). Storage-protein 2 from *B. mori* (SP2Bm) has previously been identified as one of the abundant hemolymph proteins potentially responsible for the beneficial effects observed after the use of *B. mori* hemolymph-supplemented cell culture media. By employing a dual baculovirus vector that co-expresses the SP2Bm protein alongside the GFP protein, we achieved a threefold increase in reporter protein production compared to a baculovirus vector expressing GFP alone. This study underscores the potential of hemolymph proteins sourced from various Lepidoptera species as biotechnological tools to augment baculovirus vector productivities, whether utilized as natural supplements in cell culture media or as hemolymph-derived recombinant proteins co-expressed by baculovirus vectors.

## 1. Introduction

Among the available production platforms, the baculovirus expression vector system (BEVS) stands out for several reasons. This system, utilizing insect cells combined with genetically modified baculovirus vectors, offers rapid development times and generates functional recombinant proteins akin to those produced in mammalian cells [1,2]. Developed in the 1980s, BEVS has emerged as a preferred method for generating proteins for investigational studies, diagnostic purposes, and the industrial production of subunit vaccines.

Enhancing biologics production within BEVS remains a continual technical challenge aimed at scaling up production capabilities. Prior studies have revealed that silkworm hemolymph extends host cell longevity by impeding baculovirus-induced insect cell apoptosis [3,4,5,6]. Interestingly, this effect extends to mammalian cell systems [7]. Notably, supplementing the medium with silkworm hemolymph not only prolonged cell viability, but also amplified recombinant protein production within the insect cell baculovirus system [8]. Silkworm hemolymph has shown inhibitory effects on apoptosis induced by various chemicals like actinomycin D, camptothecin, and staurosporine [6]. Among the hemolymph components responsible for these effects, the *30Hc6* encoding protein (30K) has been extensively studied [9]. Recent research has highlighted the inhibitory effects of another silkworm hemolymph protein, storage-protein 2 (SP2), which is used not only as an energy source for silkworm growth and development, but also to curtail cell apoptosis [10].

In this study, we verified that the hemolymph of another Lepidoptera species, *Trichoplusia ni* (*T. ni*), shares similar properties as a natural component in cell culture media. Furthermore, we demonstrated that *B. mori* storage-protein 2 could enhance late-stage insect cell viabilities post-infection by baculovirus vectors. Additionally, when co-expressed with a recombinant protein of interest, it augmented baculovirus-mediated productivity.

## 2. Materials and Methods

### 2.1. Insect Cells and Insect Larvae

The *Spodoptera frugiperda* Sf21 cell line was cultured adherently at 28 °C using TNM-FH culture media (PAN-Biotech GmbH, Aidenbach, Germany). This medium was supplemented with 10% heat-inactivated fetal bovine serum (PAN-Biotech GmbH, Germany), gentamicin at 50 μg/mL (Sigma, St. Louis, MO, USA (Merck)), and 1× Antibiotic-Antimycotic (Sigma, St. Louis, MO, USA (Merck)).

In contrast, the *Spodoptera frugiperda* Sf9 cell line was cultured in suspension at 28 °C and 125rpm in ESF-921 culture media (Expression Systems, Davis, CA, USA). This media was supplemented with gentamicin at 50 μg/mL (Sigma, St. Louis, MO, USA (Merck)) and 1× Antibiotic-Antimycotic (Sigma, St. Louis, MO, USA (Merck)).

*T. ni* larvae were reared under level 2 biosafety conditions, following previously described methodology [11]. Fifth instar larvae (the last instar before pupation), weighing approximately 300 mg, were utilized for all experiments. Hemolymph was collected by making a small incision in each larva’s body. Subsequently, the hemolymph was centrifuged, filtered through a 0.22-micron membrane filter, immediately frozen, and stored at −20 °C until required.

### 2.2. Hemolymph Formulation

The *T. ni* hemolymph was collected from the fifth instar larvae by punction of the insect abdomen. The collected hemolymph was heat-treated at 60 °C for 30 min, then chilled and centrifuged. The supernatant was filtered with a 0.2 m membrane filter and used for supplementing the medium. The hemolymph was incorporated into the cell culture medium at varying concentrations (1% or 2%) and employed for cultivating infected Sf21 cells. This formulated medium was applied during the infection process and sustained for different time intervals until cells were collected for analysis.

### 2.3. Cell Viability Analysis

Trypan blue staining was employed to assess cell viability. The calculation of cell viability was based on the proportion of living cells in relation to the total cell count at various time points post-infection.

### 2.4. Generation of Recombinant Baculoviruses

**Figure 1:**
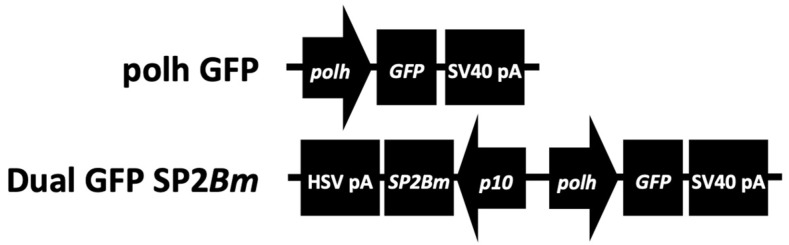
Schematic representation illustrating the genetic components of the developed baculovirus expression cassettes.

In this study, two recombinant baculoviruses were developed: one expressing storage-protein 2 (SP2Bm) and the GFP reporter protein (dual baculoviruses), and the other solely expressing GFP (Figure 1). These constructs were generated to model productivities in insect cells in the presence or absence of the SP2Bm protein. The amino acid sequence of the *B. mori* SP2 protein, obtained from the NCBI database (GenBank accession number: NP_001037590.1), served as the reference.

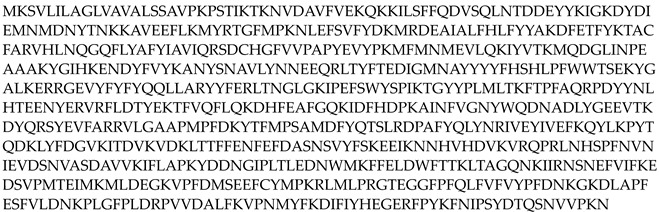



The encoding sequences for these proteins were synthesized using GenScript (Leiden, The Netherlands) services. All recombinant baculoviruses were produced by utilizing the Bac-to-Bac Baculovirus Expression System (Invitrogen^®^, Life Technologies, Carlsbad, CA, USA). Initially, donor plasmids were created using the *pFastBac*1 or *pFastBac* Dual vectors (Invitrogen). Subsequently, bacmids necessary for generating different baculoviruses were prepared in *E. coli* DH10Bac bacterial cells containing the mini Tn7-replicon. The transfection of the bacmids into Sf21 cells was conducted in order to obtain the passage I of the recombinant baculoviruses. Subsequently, the virus seeds were amplified to produce the working virus stock. The regions of the baculovirus genome containing the expression cassette and the two foreign genes underwent sequencing to confirm the integrity of the genes in the respective recombinant baculovirus.

### 2.5. Cell Infection and Recombinant Protein Analysis

Protein concentrations were determined using the Bradford method via a protein assay kit from Bio Rad Laboratories. Fluorescence analysis of GFP was conducted using 20 mg of total cell soluble protein (TSP), measured with a Tecan fluorescence plate reader (GENios) (excitation 485 nm, emission 535 nm). Furthermore, fluorescence imaging of cells infected with various baculoviruses was conducted under UV light.

## 3. Results

### 3.1. Determination of Protein Expression and Cell Viability following Baculovirus Infection under Varied Conditions

Sf21 cells underwent infection with recombinant baculoviruses at multiplicities of infection (m.o.i.) of 0.1 or 5. Post-infection, cell cultures were harvested at various time points and subjected to centrifugation for sedimentation. Subsequent steps involved the removal of supernatants and resuspension of cell pellets in PBS pH 7.2. These suspended cells were disrupted through three cycles of freezing and thawing, followed by the elimination of cellular debris via centrifugation at 23,660× *g* for 5 min at 4 °C.

**Figure 2:**
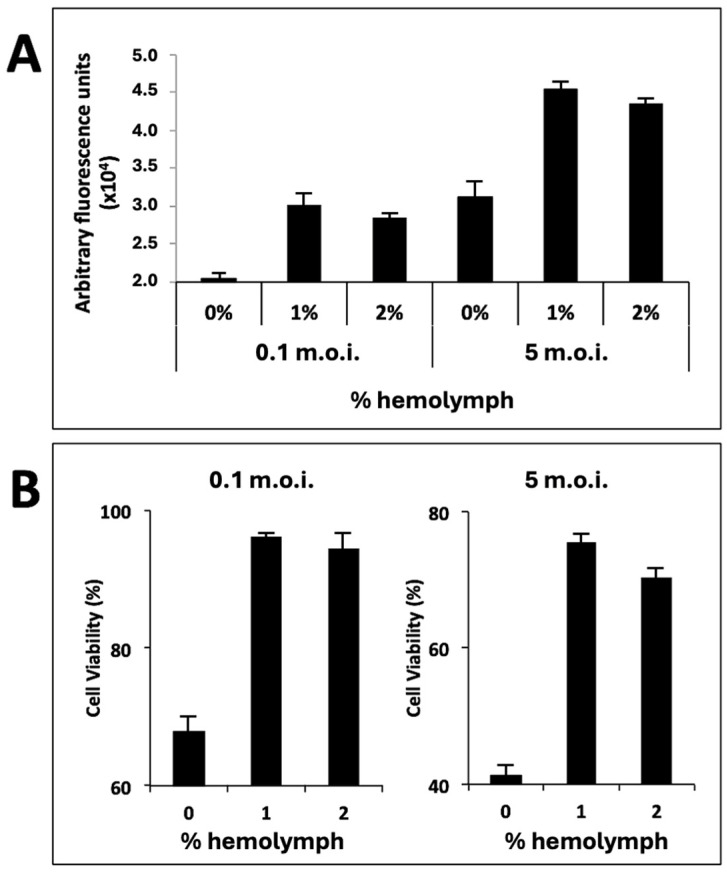
The recombinant GFP expressions and viabilities of infected Sf21 cells cultured using medium supplemented with *T. ni* hemolymph. (**A**) Fluorimetric analysis showing the arbitrary fluorescence units of cells cultured with hemolymph-supplemented medium and infected at 96 hpi with recombinant baculovirus expressing GFP under the control of the *polyhedrin* promoter at m.o.i. levels of 0.1 or 5. The bars represent the means of three independent experiments. (**B**) The analysis of cell viabilities at 96 hpi in the same experiment is described in panel A. The bars also represent the means of the three independent experiments.

When cells were cultivated with 1% or 2% *T. ni* hemolymph, a notable enhancement of GFP expression—by approximately 150%—was observed post-infection, regardless of the m.o.i. used (Figure 2A). Additionally, cell viability at late post-infection times (96 h) was significantly higher in the presence of this hemolymph (Figure 2B).

### 3.2. Analysis of the Effects on Protein Expression and Cell Viability after Using Recombinant Baculoviruses Co-Expressing the Storage-Protein 2 from B. mori (SP2Bm)

Previously, the SP2Bm protein was identified as an anti-apoptotic protein in both insect and mammalian cells [11]. In this study, our aim was to demonstrate the potential beneficial effects of this insect-derived protein on cell viability and foreign protein expression in the baculovirus expression system. For this purpose, we created a dual baculovirus co-expressing both the SP2Bm and GFP proteins, and compared its cell viabilities and GFP expression yields with a recombinant baculovirus expressing only GFP under the control of the same promoter (*polyhedrin*).

**Figure 3:**
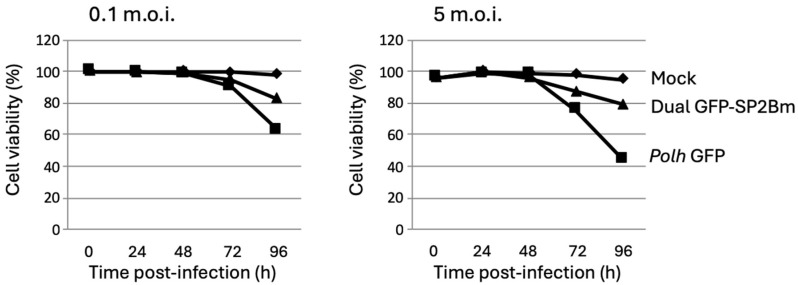
Analysis of Sf9 insect cell viability in suspension culture infected with recombinant baculoviruses expressing GFP or GFP + SP2Bm proteins at m.o.i. levels of 0.1 or 5, up to 96 hpi.

When insect cells were infected at m.o.i. of 0.1 or 5 with the dual baculovirus co-expressing the SP2Bm protein, insect cell viabilities notably improved, especially at late post-infection times (96 h), similar to the effects observed after using hemolymph derived from *T. ni* (Figure 3). This effect was more pronounced at a higher multiplicity of infection, resulting in a nearly two-fold increase in the percentage of surviving cells compared to when the SP2Bm protein was absent.

**Figure 4:**
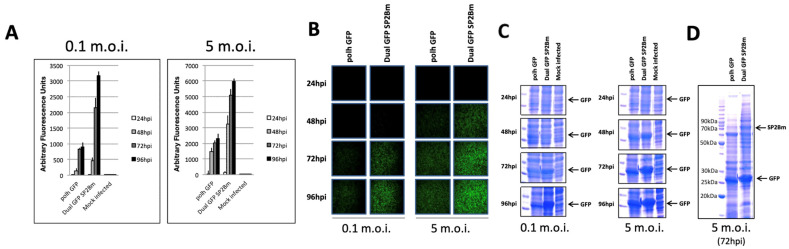
The recombinant GFP expression in Sf21 insect cells infected with recombinant baculoviruses expressing GFP or GFP + SP2Bm proteins. (**A**) Fluorimetric analysis displaying the arbitrary fluorescence units of cells infected at 96 hpi with the baculoviruses at m.o.i. levels of 0.1 or 5. The bars represent the means of three independent experiments. (**B**) The representative visual fluorescence observed under UV light after recombinant GFP expression in Sf21 insect cells infected at m.o.i. levels of 0.1 or 5 with the recombinant baculoviruses (magnification 10×). (**C**) The Coomassie blue staining of SDS-PAGE gels showing the resolved proteins of cell extracts in both experimental infection conditions. (**D**) The expression of SP2BM protein in insect cells infected at a m.o.i. of 5 and at 72 hpi.

Regarding foreign protein expression (GFP), the presence of the SP2Bm protein facilitated its expression by approximately 2–3 times at late post-infection times (72–96 h), as determined by fluorimetric analysis (Figure 4A). This increase in productivity surpassed the enhancement observed in cultures supplemented with *T. ni* insect hemolymph. These findings suggest the potential of insect-derived proteins in enhancing productivity using baculovirus vector expression systems, highlighting significant biotechnological applications.

These effects were visibly evident under a fluorescence microscope while observing infected cells at different post-infection times at both 0.1 and 5 m.o.i. (Figure 4B), as well as during SDS-PAGE analysis of cell protein extracts stained with Coomassie blue (Figure 4C).

### 3.3. T. ni-Derived Proteins with Similarities to SP2Bm from B. mori

**Figure 5:**
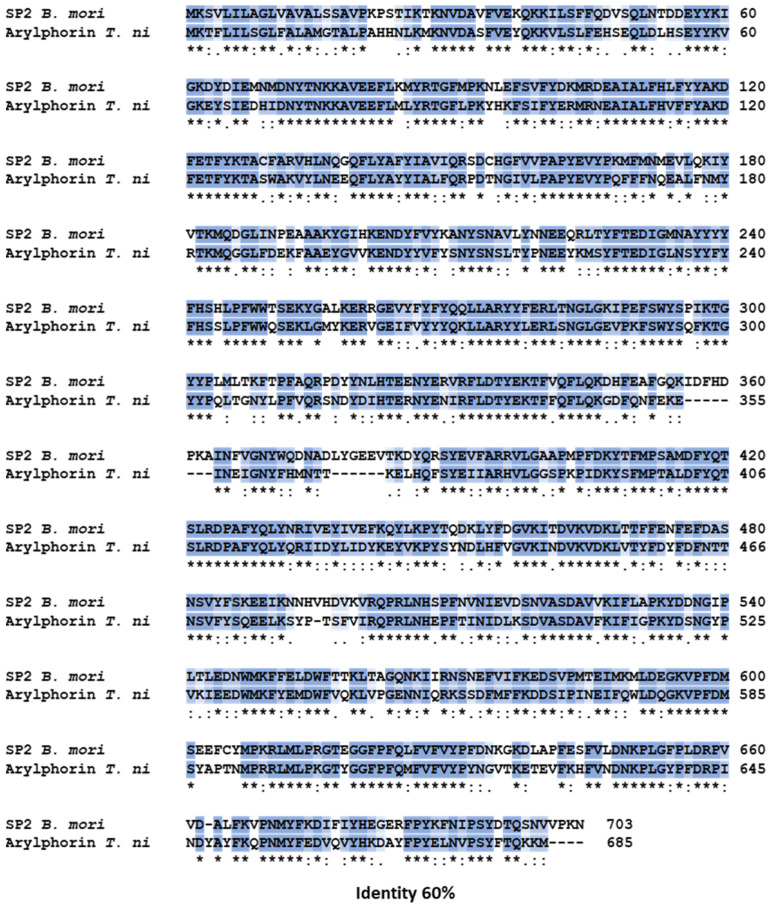
The alignment of the SP2Bm protein with the Arylphorin protein of *T.ni* (GenBank accession number: XP_026725423.1). Dark blue (*) identical sequence. Light blue (:) conservative mutation.

Given the similar positive effects observed in *T. ni* and *B.* hemolymph, we sought similarities between proteins from *T. ni* and the SP2Bm protein. We compared available sequences of *T. ni* proteins annotated in databases with the SP2Bm protein. The analysis focused on the abundant storage proteins Arylphorin, AJSP-1, BJSP-1, and BJSP-2, comparing them to SP2Bm. The results showed that only *T. ni* Arylphorin exhibits similarities with SP2Bm protein that are higher than 60% (Figure 5). This implies that Arylphorin may play a crucial role in the effects on cell viability and protein production observed in *T. ni* hemolymph, described herein. Additionally, this protein could potentially serve biotechnological purposes by functioning as an anti-apoptotic molecule similar to the SP2Bm protein. Future experiments will aim to establish the function of *T. ni*-derived Arylphorin in this context.

## 4. Discussion

Insect cell viability during infection typically declines when using conventional cell culture mediums. However, in contrast, cell viability remained consistently high for up to 6 days after baculovirus infection when the medium was supplemented with 1% or 2% *T. ni* hemolymph. This outcome mirrored the effects observed when using a medium supplemented with 5% silkworm hemolymph [3,4]. Furthermore, it was observed that DNA fragmentation did not occur within the initial 3 days, but surfaced thereafter in the medium lacking silkworm hemolymph [6]. Although we did not specifically investigate late-stage apoptosis inhibition in cells exposed to *T. ni*-derived hemolymph, it is plausible that the observed phenomena could be explained through a similar mechanism. Silkworm hemolymph has proven instrumental in efficiently producing recombinant proteins within the insect cell baculovirus system, as the viability of host cells is pivotal for both the replication of baculovirus DNA containing a recombinant gene and the expression of the cloned gene [3]. Similarly, this natural compound could hold promise for mammalian cell cultures, as silkworm hemolymph has shown beneficial effects on culture viability [8].

To our knowledge, hemolymph from different Lepidoptera species has not been previously explored within the context of BEVS (Baculovirus Expression Vector System). In our study, we tested *T. ni*-derived hemolymph under conditions similar to those used for silkworm hemolymph. Our findings indicate that *T. ni*-derived hemolymph contains compounds that increase cell viability post-baculovirus infection, even at lower concentrations than silkworm hemolymph [3]. This suggests the presence of proteins in *T. ni* hemolymph that possess properties akin to those of the previously described storage proteins found in silkworm hemolymph [8,9]. An analysis aligning several proteins with similar functional activities in the two Lepidoptera species revealed that *T. ni* Arylphorin protein shares similarities exceeding 60% with SP2Bm protein from *B. mori* [12], suggesting a potential similar role in enhancing cell viability post-baculovirus infection in insect cells. Investigations to confirm this hypothesis are underway.

Given the correlation between apoptosis inhibition, increased cell viability post-baculovirus infection, and enhanced recombinant protein productivity [9], we explored this effect using media supplemented with *T. ni* hemolymph. Our results demonstrated a 1.5-fold increase in the productivity of the reporter protein GFP when used at concentrations of 1–2% in the cell culture medium, aligning with observations made regarding silkworm hemolymph.

The *B. mori* SP2Bm protein seems to be one of the components in silkworm hemolymph that inhibits baculovirus-induced apoptosis and enhances cell viability in insect cells. It also functions as an apoptosis inhibitor in mammalian cells [7,10]. We investigated the possibility of co-expressing the SP2Bm protein alongside a protein of interest within the same baculovirus vector to enhance both cell viability and productivity. This dual baculovirus exhibited a significant increase in productivity compared to a baculovirus expressing only the protein of interest. Additionally, cell cultures infected at late stages with the dual baculovirus showed increased cell viability compared to those infected with the vector expressing the recombinant protein in the absence of the SP2Bm protein.

In summary, this study highlights the potential of hemolymph proteins sourced from various Lepidoptera species as a biotechnological resource. While *B. mori* hemolymph has been recognized as a source of valuable components for industrial biologics manufacturing via the baculovirus expression system, our findings show that other Lepidoptera species, like *T. ni*, might offer natural compounds that can boost productivity at laboratory or industrial scales. Moreover, we demonstrated that the efficacy of co-expressing at least one component of *B. mori* hemolymph, the SP2Bm protein, presents an excellent method for improving baculovirus vectors, without the necessity of using Lepidoptera-derived hemolymph. These findings advocate for the continued exploration of insect-derived compounds that could enhance productivity in bioreactor-based manufacturing across different cell platforms.

## Data Availability

All data created in this study have already been shown in the manuscript. No more new data are available.
